# Dysplastic Transformation in Sporadic Fundic Gland Polyps: Prevalence, Clinical and Endoscopic Characteristics in an Asian Cohort

**DOI:** 10.3390/cancers18040616

**Published:** 2026-02-13

**Authors:** Ming-Jung Meng, Tsung-Hsing Chen, Shih-Chiang Huang

**Affiliations:** 1 Department of Gastroenterology and Hepatology, Linkou Chang Gung Memorial Hospital, Taoyuan 33305, Taiwan; r1231234562002@gmail.com; 2 College of Medicine, Chang Gung University, No. 5, Fu-Hsin St., Guei-Shan, Taoyuan 33305, Taiwan; 3 Department of Anatomic Pathology, College of Medicine, Linkou Chang Gung Memorial Hospital, Chang Gung University, Taoyuan 33305, Taiwan; ab86401112@cgmh.org.tw

**Keywords:** gastric polyps, fundic gland polyps, proton pump inhibitors, dysplasia, pathology archive, Asian cohort

## Abstract

Background/Objectives: Fundic gland polyps (FGPs) are the most common type of gastric polyp and have increased in prevalence in the proton pump inhibitor (PPI) era. Although traditionally considered benign, dysplasia has been described in both syndromic and sporadic FGPs; data from Asian cohorts remain limited. We evaluated the prevalence of FGPs with dysplasia (FGPD) and described associated clinical and endoscopic features in a Taiwanese tertiary-care cohort. Methods: We retrospectively searched institutional pathology archives for all gastric biopsy or polypectomy specimens diagnosed as FGP between January 2000 and December 2024 and mapped these specimens to unique patients using medical record numbers. Candidate dysplastic cases underwent slide review by gastrointestinal pathologists to confirm FGPD and grade dysplasia as low- or high-grade according to standard gastric dysplasia criteria. Cases were classified as syndromic if a hereditary polyposis syndrome was documented; otherwise, they were classified as sporadic. Clinical and endoscopic variables were abstracted from electronic medical records. Patient-level prevalence estimates among patients with FGP are reported with exact 95% confidence intervals (CIs). Results: Among 35,806 unique patients with histologically confirmed FGP, 25 FGPD cases were confirmed (21 sporadic, 4 syndromic). The patient-level prevalence of sporadic FGPD was 0.059% (21/35,806; 95% CI: 0.036–0.090%). Among sporadic cases, dysplasia was low-grade in 19 (90.5%) and high-grade in 2 (9.5%). Sporadic cases occurred at a median age of 48 years (interquartile range [IQR]: 37–63.5 years), and 57.1% were female. Documented PPI exposure before the index FGPD endoscopy was present in 33.3% of patients (median documented duration: 36 months [IQR: 12–125]). No case had documented current *Helicobacter pylori* infection at the index evaluation. Endoscopically, sporadic FGPDs were commonly multiple, sessile, located in the gastric body/fundus, and small (median size: 0.5 cm [IQR: 0.3–0.575]). Conclusions: Sporadic FGPD was exceedingly rare in this 25-year Taiwanese cohort and was predominantly low-grade. Although typically small and body/fundus-predominant, FGPs with erythema or surface irregularity—particularly with irregular microvascular patterns on narrow-band imaging—should prompt histologic assessment to exclude dysplasia.

## Simple Summary

Fundic gland polyps (FGPs) are common gastric polyps and are usually benign, but they can rarely develop dysplasia, a precancerous epithelial change. Because data from Asian populations are limited, we reviewed a 25-year single-center pathology archive in Taiwan (2000–2024). Among 35,806 unique patients with histologically confirmed FGPs, we identified 25 cases of FGPs with dysplasia. Most were sporadic (21/25), corresponding to a patient-level prevalence of 0.059%. Sporadic cases occurred at a median age of 48 years and were slightly more common in women (57.1%). Low-grade dysplasia predominated (90.5%). One-third of patients had documented proton pump inhibitor exposure before diagnosis (median documented duration: 36 months), whereas none had documented current *Helicobacter pylori* infection at the index evaluation. Endoscopically, dysplastic FGPs were typically small (median: 0.5 cm), often multiple, sessile, and located in the gastric body and/or fundus, frequently detected during health examinations. Subtle warning signs included erythema, surface irregularity, or focal erosion; when narrow-band imaging was used, irregular or dilated microvascular patterns were described. These clinical and endoscopic characteristics may help clinicians recognize when an FGP warrants targeted biopsy or complete endoscopic resection, while highlighting the need for prospective studies with appropriate comparator groups.

## 1. Introduction

Fundic gland polyps (FGPs) are the most common type of gastric polyp and account for approximately 80% of all gastric polyps [1]. During the 1990s, the prevalence of FGPs among patients undergoing esophagogastroduodenoscopy (EGD) was reported to range from 0.21% to 2.0% [2,3,4]. In the era of widespread proton pump inhibitor (PPI) use, multiple studies have reported increases in the detection of FGPs, with prevalence estimates since 2000 ranging from 5.9% to 30.3% [5,6].

FGPs can be classified as sporadic or syndromic. Syndromic FGPs occur in the context of hereditary polyposis syndromes, including familial adenomatous polyposis (FAP), attenuated FAP, gastric adenocarcinoma and proximal polyposis of the stomach (GAPPS), and MUTYH-associated polyposis (MAP) [7,8,9,10]. Sporadic FGPs are typically observed in middle-aged women, whereas syndromic FGPs tend to occur at younger ages [11].

Historically, sporadic FGPs have been regarded as lesions with minimal malignant potential. In contrast, syndromic FGPs can exhibit low- or high-grade dysplasia and, rarely, adenocarcinoma. More recently, dysplasia has also been reported in sporadic FGPs, raising questions relevant to cancer prevention and early neoplasia detection: when should an FGP be biopsied or resected, and how should patients be counseled regarding the risk of neoplastic transformation? [11,12,13,14].

Across endoscopic series, FGPs are frequently incidental findings detected during evaluation for dyspepsia or reflux, or as part of health examinations. The relative distribution of gastric polyp subtypes differs by geographic region and over time, reflecting changes in background gastritis, *Helicobacter pylori* prevalence, and patterns of acid suppression. Large endoscopic cohorts from different regions have described temporal shifts in the gastric polyp spectrum, with FGPs becoming proportionally more common as *H. pylori* prevalence declines and PPI use increases [15,16,17,18]. In routine practice, this epidemiologic shift translates into an increasing number of patients with multiple small, smooth-surfaced, translucent polyps in the gastric body/fundus, a pattern that is typically consistent with FGPs [16].

Observational studies have reported an association between long-term PPI therapy and an increased prevalence of FGPs, and a systematic review and meta-analysis likewise supports this relationship, although residual confounding and indication bias cannot be fully excluded [19,20]. Conversely, FGPs are less frequent in the setting of active *H. pylori* infection, suggesting that the inflammatory and acid-secretory milieu may influence FGP development [6].

At the molecular level, sporadic FGPs often show activation of the Wnt signaling pathway through somatic CTNNB1 (β-catenin) mutations, whereas syndromic FGPs in FAP typically arise in the setting of germline APC alterations with secondary pathway hits [21,22]. Dysplasia within FGPs is therefore biologically plausible, but its clinical expression appears strongly dependent on underlying predisposition. FAP-associated FGPs frequently show architectural complexity and foveolar dysplasia [12,23], whereas dysplasia in sporadic FGPs has been reported only occasionally, most often as low-grade dysplasia and rarely as carcinoma [11,14,24,25,26].

Because dysplasia represents a pre-neoplastic endpoint and published data in Asian populations are scarce, we performed a retrospective, pathology archive-based study at a Taiwanese tertiary center spanning 2000–2024. Our primary aim was to estimate the patient-level prevalence of sporadic fundic gland polyps with dysplasia (FGPD) among patients with pathologically confirmed FGP. Secondary aims were to describe clinical characteristics, medication exposures, endoscopic morphology (including image-enhanced endoscopic patterns when available), and dysplasia grade at diagnosis. In patients with serial pathology, we also report the clinically observed interval between the first pathologically confirmed non-dysplastic FGP and the first FGPD. Syndromic FGPD cases were also identified and described for context; given the small number of syndromic cases, no inferential comparisons were planned.

By defining the prevalence and clinicopathologic correlates of FGPD in a large Taiwanese cohort, this study aims to address an important evidence gap and provide clinically actionable information to support risk stratification, guide biopsy or resection decisions, and improve patient counseling—thereby informing more consistent, prevention-oriented care and establishing a foundation for future prospective studies and region-specific practice recommendations.

## 2. Materials and Methods

### 2.1. Study Design, Setting, and Ethics

This retrospective, single-center observational study was conducted at Chang Gung Memorial Hospital, Linkou branch (Taoyuan, Taiwan), a tertiary referral center. The study was approved by the Institutional Review Board of Chang Gung Memorial Hospital, Linkou (approval No. 202500745B0; approval date 2 June 2025) and conducted in accordance with the Declaration of Helsinki.

### 2.2. Case Identification and Units of Analysis

We queried the institutional pathology archive to identify all gastric biopsy or polypectomy specimens with a diagnosis of FGP between 1 January 2000 and 31 December 2024. Specimens were linked to unique patients using medical record numbers; the total denominator (35,806) represents unique patients with at least one histologically confirmed FGP identified during esophagogastroduodenoscopy in the study period. For patients with multiple FGP diagnoses over time, the earliest pathologically confirmed FGP was considered the index FGP for interval calculations.

Prevalence was calculated at the patient level (number of patients with FGPD divided by number of patients with FGP). Clinical and endoscopic analyses were also performed at the patient level, using the first (index) FGPD diagnosis per patient. Because multiple polyps were often present and endoscopists sometimes submitted pooled biopsies, polyp-level denominators (e.g., total number of polyps present or sampled per endoscopy) could not be consistently reconstructed; therefore, endoscopic characteristics are reported descriptively.

### 2.3. Definitions and Eligibility Criteria

FGPD was defined as dysplasia arising within a histologically confirmed FGP. Dysplasia was graded as low grade or high grade in accordance with the World Health Organization (WHO) classification of tumors of the digestive system [27]. Specimens interpreted as “indefinite for dysplasia” were not included as FGPD. Cases were classified as syndromic if the patient had a documented diagnosis of FAP, attenuated FAP, or MAP in the medical record at or before the index endoscopy; cases without such history were classified as sporadic.

### 2.4. Pathology Review and Adjudication

All candidate FGPD cases were re-reviewed on hematoxylin and eosin (H&E)-stained sections by board-certified gastrointestinal pathologists to confirm the diagnosis and dysplasia grade. When grading was uncertain, cases were reviewed in a consensus conference to reach a final diagnosis. Specimen type (biopsy vs. polypectomy) and the presence of significant artifact (e.g., crush/cautery) were recorded when documented; specimens that were insufficient for reliable grading were excluded.

### 2.5. Clinical, Medication, and Endoscopic Data

Clinical data were abstracted from electronic medical records, including age, sex, tobacco and alcohol use, indications for EGD, history of malignancy, and follow-up endoscopy when available. Malignancy history was defined as any malignancy diagnosed prior to the index FGPD pathology date. For patients with documented PPI exposure, the clinician-recorded indication for PPI therapy was abstracted when available.

PPI exposure was ascertained from prescription records and medication lists/clinical notes. Duration was calculated as the cumulative months of documented PPI use prior to the index FGPD endoscopy when start and stop dates were available. Over-the-counter use and external prescriptions may not have been captured. Other acid-suppressive agents (e.g., H2-receptor antagonists) were not systematically captured and were therefore not analyzed.

*Helicobacter pylori* status was abstracted from pathology reports and medical records around the index endoscopic evaluation, including histologic assessment of gastric biopsies and other test results when documented (e.g., rapid urease test, urea breath test, or stool antigen). Testing was not uniform across the 25-year period.

Endoscopic characteristics (location, size, number, and morphology) were extracted from endoscopy reports at the time of FGPD diagnosis. When multiple FGPs were present, size was recorded as the largest reported diameter. Narrow-band imaging (NBI) findings were recorded when NBI was performed and described in the report or images were available.

### 2.6. Follow-Up and Time Interval Definitions

Follow-up duration was calculated from the index FGPD diagnosis date to the last available endoscopic follow-up or clinical record. The interval from FGP to FGPD was defined as the time between the first pathologically confirmed diagnosis of non-dysplastic FGP and the first pathologically confirmed diagnosis of FGPD in the same patient. This interval should not be interpreted as a definitive biological progression time because repeat sampling was performed at the clinician’s discretion.

### 2.7. Statistical Analysis

Continuous variables are presented as medians with interquartile ranges (IQRs), and categorical variables as counts and percentages. Patient-level prevalence estimates among patients with FGP are reported with exact (Clopper–Pearson) 95% confidence intervals. Given the small number of syndromic cases, analyses were descriptive, and no formal comparisons between sporadic and syndromic groups were performed. Analyses were conducted using IBM SPSS Statistics version 26.0 (IBM Corp., Armonk, NY, USA).

## 3. Results

### 3.1. Study Flow, Prevalence, and Patient Characteristics

A total of 35,806 unique patients had at least one pathologically confirmed diagnosis of FGP during the study period (January 2000 to December 2024). Among these patients, 25 met criteria for FGPD after pathologist review: 21 sporadic cases and 4 syndromic cases. The patient-level prevalence of all FGPD (sporadic plus syndromic) was 0.070% [95% CI: 0.045–0.103%]. The patient-level prevalence of sporadic FGPD was 0.059% [95% CI: 0.036–0.090%]. Syndromic FGPD accounted for 0.011% [95% CI: 0.003–0.029%].

Clinical characteristics of sporadic FGPD are summarized in Table 1. The median age at index FGPD diagnosis was 48 years [IQR: 37–63.5], and 57.1% of patients were female. Dysplasia was low grade in 19 patients (90.5%) and high grade in 2 patients (9.5%). A history of malignancy diagnosed prior to FGPD was documented in five patients (23.8%), including three stage I colorectal cancers and two stage I gastric cancers, all treated with surgical resection; no malignant transformation of fundic gland polyps was identified. Documented PPI exposure was present in seven patients (33.3%), with a median documented duration of 36 months [IQR: 12–125]. No sporadic FGPD patient had documented current *H. pylori* infection at the index evaluation.

Index EGD leading to FGPD diagnosis was most commonly performed as part of a health examination (52.4%), followed by dyspepsia (23.8%) and acid regurgitation (14.3%); other indications accounted for 9.5%. The median follow-up duration was 25 months [IQR: 8–29]. Among patients with a prior non-dysplastic FGP diagnosis in our archive, the median interval from first FGP to first FGPD diagnosis was 10 months [IQR: 2–21.7]. Because follow-up EGD was performed at the discretion of the treating clinician rather than at fixed intervals, this measure should be interpreted as a clinically observed interval rather than a definitive estimate of biological progression time (Table 1).

### 3.2. Endoscopic Findings

Endoscopic characteristics of sporadic FGPD are summarized in Table 2. FGPDs were most commonly located in the gastric body (57.1%) or involved both the body and fundus (28.6%). Slightly more than half of patients had multiple polyps (52.4%).

**Figure 1:**
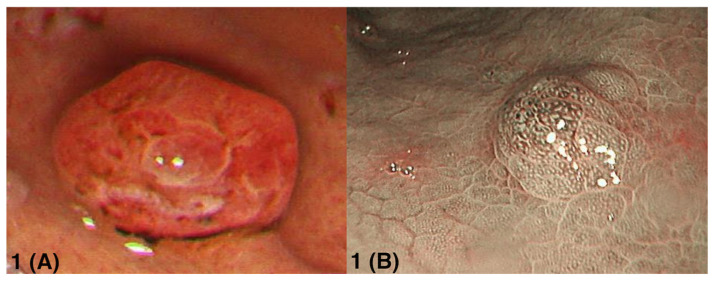
Representative endoscopic images of sporadic fundic gland polyps with dysplasia. (**A**) White-light endoscopic view of a sessile FGPD in the gastric body with an erythematous, irregular surface and superficial erosion. (**B**) Narrow-band imaging view showing irregular and dilated microvascular architecture over the lesion surface. Images are presented as representative examples; endoscopy platforms and imaging technologies evolved during the 2000–2024 study period.

Most lesions were sessile (71.4%) and small (median size: 0.5 cm [IQR: 0.3–0.575]). On white-light endoscopy, lesions frequently showed erythema and/or surface irregularity, with focal erosions or superficial ulceration described in some cases. When narrow-band imaging was performed, an irregular, dilated, or distorted microvascular pattern was described on the lesion surface (Figure 1).

In patients with multiple FGPs, pooled biopsies were commonly submitted, and the exact number of individual polyps sampled could not be consistently determined from archived records. Therefore, reported endoscopic features should be interpreted as descriptive of the endoscopic setting in which dysplasia was detected rather than as validated predictors of dysplasia.

## 4. Discussion

### 4.1. Principal Findings and Comparison with the Literature

In this 25-year, single-center pathology archive-based study from Taiwan, sporadic FGPD was exceedingly rare, with a patient-level prevalence among patients with FGP of 0.059% [95% CI: 0.036–0.090%]. This estimate is lower than dysplasia rates reported in several Western series and selected endoscopic cohorts, though direct comparison is challenging because denominators (patient-based vs. polyp-based), biopsy thresholds, and ascertainment methods differ across studies [11,12,13,14,23]. Nevertheless, the finding reinforces that dysplasia in sporadic FGPs is an uncommon endpoint even in a high-volume tertiary center.

Beyond rarity, the phenotype of sporadic FGPD in our cohort was notable for small lesion size and a predominance of low-grade dysplasia. The median largest lesion size was 0.5 cm, and 90.5% of cases were classified as low-grade dysplasia. This aligns with prior clinicopathologic series describing sporadic FGPs with low-grade dysplasia as typically small lesions with favorable short-term outcomes after endoscopic sampling or removal [23,26]. Although carcinoma arising in sporadic FGPs has been reported, it appears to be exceptionally uncommon and has generally been described in association with endoscopically atypical surface features (e.g., erythema, irregular surface, depression, or erosions) rather than the classic smooth, translucent appearance of FGPs [11,25].

In contrast to sporadic lesions, dysplasia appears to be substantially more frequent in syndromic settings, particularly in FAP, where foveolar dysplasia and architectural complexity are well recognized and may be multifocal [12,24]. This syndromic–sporadic dichotomy supports a practical clinical approach in which the detection of dysplasia in an FGP should prompt careful review of personal and family history, assessment for multiple gastric and duodenal polyps, and consideration of genetic evaluation when appropriate.

### 4.2. Pathogenesis and the Role of Acid Suppression

This section summarizes putative pathogenic contributors to sporadic fundic gland polyp dysplasia (FGPD), with particular emphasis on acid suppression, and integrates (i) epidemiologic observations, (ii) biologically plausible mucosal effects of proton pump inhibitors (PPIs), and (iii) established molecular features of sporadic fundic gland polyps (FGPs) that may permit dysplastic transformation.

#### 4.2.1. Acid Suppression and Trophic Changes in the Oxyntic Mucosa

In our cohort, PPI exposure was documented in approximately one-third of patients with sporadic FGPD, with a median recorded duration of 36 months. This observation is consistent with prior observational studies showing a higher occurrence of FGPs among long-term PPI users, with meta-analyses also supporting increased odds of FGPs in individuals receiving PPIs [19,20]. Mechanistically, sustained acid suppression may induce chronic hypergastrinemia and exert trophic effects on oxyntic glands, contributing to parietal cell alterations and cystic gland dilation—changes that could expand the pool of susceptible epithelium within which dysplasia might rarely arise [11]. Importantly, however, our study was not designed to test a causal association between PPIs and FGPD. We lacked a non-dysplastic FGP comparator group, exposure ascertainment was retrospective (with potential misclassification due to over-the-counter use or external prescriptions), and confounding by indication is likely. Moreover, indications for PPI therapy were not consistently documented over the 25-year study period and could not be incorporated as explanatory variables. Accordingly, PPI-related findings in this series should be interpreted as descriptive and hypothesis-generating rather than inferential.

#### 4.2.2. Molecular Plausibility for Dysplasia Arising Within FGPs

Beyond acid suppression, prior work indicates that activation of the Wnt signaling pathway is a central feature of sporadic FGP biology. Activating CTNNB1 (β-catenin) mutations have been identified in many sporadic FGPs, and dysplastic change has been associated with nuclear β-catenin accumulation and relatively increased epithelial turnover [14,21,22]. Taken together, these molecular observations provide biologic plausibility for dysplasia developing within FGPs, while also reinforcing that such progression likely represents an uncommon event within an otherwise indolent lesion.

#### 4.2.3. Potential Modifying Role of *Helicobacter pylori*

Notably, none of the sporadic FGPD patients in our series had documented active *H. pylori* infection at the index evaluation. This aligns with prior reports suggesting that FGP prevalence is lower in individuals with current *H. pylori* infection [6]. Nevertheless, because *H. pylori* testing practices were not uniform across the 25-year period and eradication history could not be reliably reconstructed, this finding should be interpreted cautiously.

Overall, available evidence supports a framework in which acid suppression-related trophic changes may promote the development or persistence of sporadic FGPs, while Wnt pathway activation provides a molecular substrate that may rarely permit dysplastic transformation. Future studies with prospective exposure ascertainment, standardized *H. pylori* assessment, appropriate non-dysplastic FGP comparator groups, and molecular profiling are needed to better define causal pathways and clinical risk stratification for sporadic FGPD.

### 4.3. Endoscopic Recognition and Practical Management Considerations

Although most FGPDs in our cohort were small, endoscopy reports frequently noted surface erythema, irregularity, or focal erosions, and an irregular or distorted microvascular pattern was described when narrow-band imaging was used. Prior case series similarly emphasize that dysplastic or neoplastic transformation within an otherwise typical-appearing FGP may present as a subtle surface change rather than a marked size increase [11,25]. These observations underscore the importance of careful inspection of the body/fundus mucosa in patients with multiple FGPs, particularly when image-enhanced endoscopy is available.

From a practice standpoint, there is broad consensus that typical-appearing sporadic FGPs (small, smooth, translucent polyps in the body/fundus) do not require routine biopsy, resection, or endoscopic surveillance, provided that there are no suspicious features and no clinical suspicion of a hereditary polyposis syndrome [28,29]. When suspicious features are present—such as size ≥ 1 cm, surface ulceration/erosion, depression, marked erythema, or irregular pit or vascular patterns—targeted biopsy or complete endoscopic resection is reasonable to exclude dysplasia or other polyp types [16,28,29,30,31]. In addition, because pooled biopsy submissions were common in our cohort, clinicians should be aware that pooling can limit polyp-level localization and may complicate subsequent targeted resection if dysplasia is detected in a mixed specimen.

When FGPD is confirmed, management is not standardized because population-level risk estimates and optimal follow-up intervals remain uncertain. Existing guidance for gastric epithelial dysplasia more generally emphasizes high-quality endoscopic reassessment with careful mucosal visualization, targeted biopsies, and complete resection of any visible lesion when feasible [30,31]. In this context, a pragmatic approach after sporadic FGPD diagnosis may include complete endoscopic resection of the index polyp (if not already removed), careful inspection of the surrounding oxyntic mucosa for additional atypical lesions, and individualized follow-up that accounts for dysplasia grade, endoscopic completeness, and patient risk factors.

### 4.4. Interpretation of the Clinically Observed FGP-to-FGPD Interval

A distinctive feature of our dataset is the ability to describe the interval between the first pathologically confirmed non-dysplastic FGP and the first FGPD in the same patient when serial pathology was available. Importantly, follow-up EGD in this cohort was performed at the discretion of the treating clinician rather than according to a fixed surveillance protocol. Therefore, the reported interval (median: 10 months) should be interpreted as a descriptive, clinically observed interval that is influenced by surveillance intensity, indications for repeat endoscopy, and sampling decisions, rather than as a definitive estimate of the biological time required for dysplastic transformation.

### 4.5. Strengths and Limitations

This study has several strengths, including a large patient-level denominator over a 25-year period, the use of a centralized pathology archive with case re-review by gastrointestinal pathologists, and the inclusion of real-world endoscopic practice patterns. Nevertheless, important limitations affect interpretation. First, although the prevalence denominator was patient-based, polyp-level denominators were not consistently available; pooled biopsy submissions and changes in documentation prevented reliable reconstruction of the number of individual polyps present or sampled at endoscopy. Second, verification bias is likely because dysplasia can only be detected in sampled tissue and endoscopists may have preferentially biopsied lesions with atypical appearances. Third, the long study timeframe introduces potential era effects, including temporal drift in endoscopic imaging platforms, biopsy practices, electronic medical record completeness, and diagnostic thresholds. Fourth, *H. pylori* testing was not uniform, and the absence of documented infection cannot distinguish between never-infected patients, previously infected and cured patients. Finally, the small number of FGPD cases, particularly syndromic cases, precluded robust comparative analyses and limited our ability to model risk factors.

### 4.6. Future Directions

Future prospective multicenter studies that include a well-defined non-dysplastic FGP comparator group, standardized sampling protocols, and centralized pathology review are needed to quantify risk factors and clarify the natural history of sporadic FGPD. Incorporating molecular profiling (e.g., Wnt-pathway alterations, mismatch repair status, and broader genomic features) could help distinguish sporadic lesions with truly neoplastic potential from indolent polyps that harbor focal, limited dysplastic change. Finally, studies that integrate standardized endoscopic imaging (including magnifying image-enhanced endoscopy) with pathology and outcomes would be particularly valuable for developing evidence-based thresholds for biopsy, resection, and follow-up after FGPD diagnosis.

## 5. Conclusions

Sporadic fundic gland polyps with dysplasia were extremely uncommon in this Taiwanese pathology archive. When detected, lesions were typically small sessile polyps in the gastric body/fundus and could exhibit erythema or surface irregularity, with irregular microvascular patterns on narrow-band imaging when available. These findings support a pragmatic approach in which typical-appearing sporadic FGPs are managed conservatively, while polyps with atypical surface change undergo targeted sampling or complete resection and patients are assessed for potential syndromic contexts when appropriate. Given the retrospective design, limited ability to reconstruct polyp-level sampling denominators, and absence of a non-dysplastic comparator group, our findings should be interpreted as descriptive and hypothesis-generating rather than evidence of causal associations. The clinically observed interval from first FGP to first FGPD reflects variable surveillance intensity and should not be interpreted as a definitive measure of biological progression time. Prospective studies are warranted to define optimal management and follow-up strategies.

## Figures and Tables

**Table 1:** Clinical characteristics of sporadic fundic gland polyps with dysplasia.

	n = 21
Age (years), median [IQR]	48 [37–63.5]
Female, n (%)	12 (57.1)
Smoking, n (%)	2 (9.5)
Alcohol use, n (%)	1 (4.8)
History of malignancy, n (%)	5 (23.8) †
PPI use before index FGPD, n (%)	7 (33.3)
Documented PPI duration (months), median [IQR]	36 [12–125]
Documented *H. pylori* infection at index evaluation, n (%)	0 (0)
Low-grade dysplasia, n (%)	19 (90.5)
High-grade dysplasia, n (%)	2 (9.5)
Indication for first EGD	
Dyspepsia	5 (23.8)
Acid regurgitation	3 (14.3)
Health examination	11 (52.4)
Other	2 (9.5)
Follow-up duration (months), median [IQR]	25 [8–29]
Time from first FGP to first FGPD (months), median [IQR]	10 [2–21.7]

† Malignancy history refers to prior malignancies diagnosed before FGPD (stage I colorectal cancer, n = 3; stage I gastric cancer, n = 2); all patients had undergone surgical resection. No malignant transformation of fundic gland polyps was observed. Abbreviations: EGD, esophagogastroduodenoscopy; FGP, fundic gland polyp; FGPD, fundic gland polyp with dysplasia; *H. pylori*, *Helicobacter pylori*; IQR, interquartile range; PPI, proton pump inhibitor.

**Table 2:** Endoscopic characteristics of sporadic fundic gland polyps with dysplasia.

	n = 21
Location	
Gastric fundus, n (%)	2 (9.5)
Gastric body, n (%)	12 (57.1)
Gastric cardia, n (%)	1 (4.8)
Gastric fundus and body, n (%)	6 (28.6)
Number of polyps at index endoscopy	
Single, n (%)	10 (47.6)
Multiple, n (%)	11 (52.4)
Largest polyp size (cm), median [IQR]	0.5 [0.3–0.575]
Morphology	
Sessile, n (%)	15 (71.4)
Pedunculated, n (%)	4 (19.0)
Other/unspecified, n (%)	2 (9.5)
Associated endoscopic findings *	
Superficial gastritis, n (%)	9 (42.9)
Chronic gastritis, n (%)	7 (33.3)
Gastroesophageal reflux disease, n (%)	8 (38.1)
Gastric or duodenal ulcers, n (%)	6 (28.6)

* Findings were extracted from index endoscopy reports and were not mutually exclusive. Abbreviations: IQR, interquartile range.

## Data Availability

The corresponding author will share the data underlying this article upon reasonable request.
